# In Silico Identification of Potential Clovibactin-like Antibiotics Binding to Unique Cell Wall Precursors in Diverse Gram-Positive Bacterial Strains

**DOI:** 10.3390/ijms26041724

**Published:** 2025-02-18

**Authors:** Olimpo Sierra-Hernandez, Oscar Saurith-Coronell, Juan Rodríguez-Macías, Edgar Márquez, José Ramón Mora, José L. Paz, Maryury Flores-Sumoza, Adel Mendoza-Mendoza, Virginia Flores-Morales, Yovani Marrero-Ponce, Stephen J. Barigye, Felix Martinez-Rios

**Affiliations:** 1 Departamento de Medicina, División Ciencias de la Salud, Universidad del Norte, Km 5, Vía Puerto Colombia, Puerto Colombia 081007, Colombia; olimpos@uninorte.edu.co (O.S.-H.); osaurith@uninorte.edu.co (O.S.-C.); 2 Grupo de Investigaciones en Química y Biología, Departamento de Química y Biología, Facultad de Ciencias Básicas, Universidad del Norte, Carrera 51B, Km 5, Vía Puerto Colombia, Barranquilla 081007, Colombia; 3 Facultad de Ciencias de la Salud, Exactas y Naturales, Universidad Libre, Barranquilla 080001, Colombia; juand.rodriguezm@unilibre.edu.co; 4 Grupo de Química Computacional y Teórica (QCT-USFQ), Departamento de Ingeniería Química, Universidad San Francisco de Quito, Diego de Robles y Vía Interoceánica, Quito 170901, Ecuador; jrmora@usfq.edu.ec; 5 Departamento Académico de Química Inorgánica, Facultad de Química e Ingeniería Química, Universidad Nacional Mayor de San Marcos, Lima 15081, Peru; jpazr@unmsm.edu.pe; 6 Programa de Química y Farmacia, Facultad de Ciencias Básicas y Biomédicas, Universidad Simón Bolívar, Carrera 59 N° 59-65, Barranquilla 080002, Colombia; maryury.flores@unisimon.edu.co; 7 Programa de Ingeniería Industrial, Universidad del Atlántico, Barranquilla 080001, Colombia; adelmendoza@uniatlantico.edu.co; 8 Laboratorio de Síntesis Asimétrica y Bioenergética (LSAyB), Ingeniería Química (UACQ), Universidad Autónoma de Zacatecas, Campus XXI Km 6 Carr. Zac-Gdl, Zacatecas 98160, Mexico; virginia.flores@uaz.edu.mx; 9 Facultad de Ingeniería, Universidad Panamericana, Augusto Rodin No. 498, Insurgentes Mixcoac, Benito Juárez, Ciudad de México 03920, Mexico; ymarrero@usfq.edu.ec (Y.M.-P.); felix.martinez@up.edu.mx (F.M.-R.); 10 Grupo de Medicina Molecular y Traslacional (MeM&T), Colegio de Ciencias de la Salud (COCSA), Escuela de Medicina, Edificio de Especialidades Médicas, Diego de Robles y Vía Interoceánica, Universidad San Francisco de Quito (USFQ), Quito 170157, Ecuador; 11 Departamento de Química Física Aplicada, Facultad de Ciencias, Universidad Autónoma de Madrid (UAM), 28049 Madrid, Spain; sjbarigye@gmail.com

**Keywords:** clovibactin analogs, in silico, lipid II, molecular dynamics, ADME-Tox, cell wall synthesis, solvation energy, ligand-receptor interactions, binding affinity properties

## Abstract

The rise in multidrug-resistant bacteria highlights the critical need for novel antibiotics. This study explores clovibactin-like compounds as potential therapeutic agents targeting lipid II, a crucial component in bacterial cell wall synthesis, using in silico techniques. A total of 2624 clovibactin analogs were sourced from the PubChem database and screened using ProTox 3.0 software based on their ADME-Tox properties, prioritizing candidates with favorable pharmacokinetic profiles and minimal toxicity. Molecular docking protocols were then employed to assess the binding interactions of the selected compounds with lipid II. Our analysis identified Compound 22 as a particularly promising candidate, exhibiting strong binding affinity, stable complex formation, and high selectivity for the target. Binding energy analysis, conducted via molecular dynamics simulations, revealed a highly negative value of −25.50 kcal/mol for Compound 22, surpassing that of clovibactin and underscoring its potential efficacy. In addition, Compound 22 was prioritized due to its exceptional binding affinity to lipid II and its favorable ADME-Tox properties, suggesting a lower likelihood of adverse effects. These characteristics position Compound 22 as a promising candidate for further pharmacological development. While our computational results are encouraging, experimental validation is essential to confirm the efficacy and safety of these compounds. This study not only advances our understanding of clovibactin analogs but also contributes to the ongoing efforts to combat antimicrobial resistance through innovative antibiotic development.

## 1. Introduction

Antimicrobial resistance presents a formidable challenge to global public health due to its rapid and significant impact. With an alarming number of annual deaths attributed to this issue, projections indicate that by the year 2050, approximately 10 million deaths will occur as a direct consequence. This phenomenon transcends territorial boundaries, generating significant repercussions in the social, healthcare, and economic sectors [1].

The selective pressure associated with the improper use of antibiotics has exponentially facilitated the development of antimicrobial resistance. This resistance has evolved through various mechanisms contributing to its spread, such as horizontal gene transfer and the acquisition of resistance factors from the environment. This phenomenon is particularly evident in environments like the human microbiota and water sources, highlighting the critical relationship between humans and natural ecosystems. The close contact between human and animal waste and the significant release of antibiotic residues into the environment increases the interaction between bacteria and these substances. This interaction provides the necessary conditions for bacteria to develop resistance to antibiotics [2,3,4,5].

The increasing prevalence of resistant bacteria, such as methicillin-resistant *Staphylococcus aureus* (MRSA) and vancomycin-resistant enterococci (VRE), poses a critical public health challenge [6]. MRSA, with resistance to methicillin and intermediate susceptibility to vancomycin (VAN), causes serious infections like pneumonia, osteomyelitis, and endocarditis [7,8,9]. VRE, including *Enterococcus faecium* and *Enterococcus faecalis*, present resistance to VAN, complicating the treatment of urinary tract infections, intra-abdominal infections, and endocarditis [7,9]

*Streptococcus pneumoniae* (SP), which is resistant to macrolides, affects the treatment of pneumonia, meningitis, and otitis media [10], while *Helicobacter pylori* (HP), which is resistant to clarithromycin, is associated with gastric ulcers and chronic gastritis [11,12]. Simultaneous resistance in these bacteria highlights the limitations of current therapies. The rapid evolution of resistance mechanisms has outpaced the development of new antimicrobial treatments. This gap underscores the need for innovative approaches and sustained investment in research to counter the growing threat of multi-resistant bacteria [13,14,15].

So far, the discovery of new antibiotics has involved both traditional and modern methods. Scientists have screened natural products, mined genomes, used metagenomics, and applied artificial intelligence to predict promising antibiotic candidates. Despite these efforts, this process is incredibly challenging. Developing a new antibiotic can take 10–15 years and cost over $1 billion, with many candidates failing during the rigorous testing phases [16,17]. This challenge is made even more difficult by the rapid emergence of resistant strains. Currently, there are only a few potential antibiotics in Phase III clinical trials, highlighting the gap between the slow pace of antibiotic development and the quick appearance of resistant bacteria [18,19].

To address this critical gap, in silico tools have become invaluable in the search for new antibiotics. These computational methods, like molecular docking, virtual screening, and machine learning, allow scientists to quickly sift through huge compound libraries and predict how they might interact with bacterial targets [20,21].

The benefits of these tools are significant: they reduce the time and cost of drug discovery, improve the accuracy of target identification, and help optimize drug candidates before they move into expensive experimental phases. For example, the antibiotic halicin was discovered using artificial intelligence, showcasing the potential of these methods to find new compounds that traditional approaches might miss [22]. Additionally, in silico techniques have been used to design teixobactin analogs that show promise against resistant bacteria [23]. By combining these advanced computational approaches with traditional experimental methods, we can speed up the development of new antibiotics, giving us a crucial edge in the fight against antimicrobial resistance [24].

One promising class of antibiotics is lantibiotics, which were first described in 1988. These are ribosomally synthesized and post-translationally modified peptides (RiPPs) with antimicrobial activity that contain meso-lanthionine and 3-methyl-lanthionine [25]. These unique modifications are incorporated into specific enzymes during biosynthesis, resulting in a highly diverse group of bioactive peptides. The discovery of lantibiotics has opened new avenues in the fight against antibiotic-resistant bacteria, with several lantibiotics demonstrating potent activity against methicillin-resistant *Staphylococcus aureus* (MRSA) and vancomycin-resistant *enterococci* (VRE) [26].

Lantibiotics work by targeting a key component of the bacterial cell wall called lipid II. By binding to this target, they block cell wall synthesis and create pores in the bacterial membrane, leading to cell death [27]. For example, nisin, a well-known lantibiotic, binds to lipid II, preventing it from being used in cell wall construction and forming pores that disrupt the membrane. Other lantibiotics that target it include mutacin, mersacidin, and lacticin 3147. This dual action makes lantibiotics highly effective against a wide range of Gram-positive bacteria [28,29,30].

The discovery of clovibactin was a breakthrough because it introduced a new class of antibiotics derived from previously uncultivated soil bacteria. Clovibactin has demonstrated strong activity against a wide range of Gram-positive pathogens, including methicillin-resistant *Staphylococcus aureus* (MRSA) and vancomycin-resistant enterococci (VRE), without any detectable resistance [30]. Structurally, clovibactin is a depsipeptide, meaning that it contains both peptide and ester bonds, very similar to lantibiotics. This unique structure enables clovibactin to bind tightly to the pyrophosphate moiety of lipid II and other crucial peptidoglycan precursors, effectively blocking cell wall synthesis. Its ability to form a flexible, hydrophobic structure that wraps around its target is similar to the binding mechanisms observed in lantibiotics [31]. This discovery not only enhances our arsenal against resistant bacteria but also underscores the untapped potential of soil microbiomes in antibiotic discovery [32,33]. By binding to lipid II, clovibactin effectively disrupts bacterial cell wall synthesis, ultimately leading to bacterial death. What sets clovibactin apart is its targeted approach; instead of affecting multiple cellular functions like many conventional antibiotics, it focuses solely on lipid II. This specificity may help mitigate the rapid emergence of resistance that often accompanies broad-spectrum antibiotics [33].

Recently, Brunicardi et al. reported a structure-activity relationship between clovibactin analogs and antibacterial activity. The authors state that alanine mutation of Leu8 and Leu7 resulted in a notable reduction in activity, which can be attributed to a decreased ability to form a “hydrophobic glove”. This glove is crucial for desolvating lipid II pyrophosphate and facilitating hydrophobic interactions with the MurNAc group. Similarly, the alanine mutation of Ser4 led to diminished activity, highlighting the importance of the serine OH group’s polar interactions with the pyrophosphate group. Furthermore, the d-alanine mutation in d-Leu2 caused a reduction in its activity, underscoring its role in membrane anchoring. Lastly, the alanine mutation of Phe1 also resulted in decreased activity, suggesting its involvement in membrane anchoring or significant hydrophobic interactions [32]. This report paves the way for the discovery of new potential candidates against Gram-positive bacteria through the modification of specific regions of clovibactin. However, to ensure efficacy, these modifications must be aligned with the type and class of molecular interactions with the target.

Regarding lipid II as an immutable target, this is not merely a strategic choice; it is a critical tactic for combating resistant bacteria. Given that lipid II is a highly conserved element present in numerous pathogenic Gram-positive bacteria, it serves as an ideal target for the development of new antibiotics [33,34,35]. By concentrating on this vital aspect of bacterial physiology, we can design drugs that are not only effective but also less prone to resistance development. This underscores the significance of our research, as we aim to identify and develop compounds akin to clovibactin, enabling us to stay ahead in the relentless fight against antibiotic-resistant infections.

The lipid nature of these targets significantly hinders the development of bacterial resistance. While these targets also include a glycoprotein component that can be modified, altering the affinity of some drugs, clovibactin’s ability to bind to the non-protein phosphate groups of multiple highly conserved targets greatly minimizes the potential for resistance development. This unique characteristic makes clovibactin a pioneering compound in the development of antibiotics with prolonged efficacy, representing a significant advancement in the fight against resistant bacterial infections [36,37].

Given the success of clovibactin, it is crucial to identify other antibiotics with similar mechanisms and structures. Targeting lipid II and other conserved elements of bacterial cell walls could offer strong protection against the rapid rise in multidrug-resistant strains. By focusing on antibiotics that bind to these essential and highly conserved components, we can slow down the race between developing new antibiotics and the emergence of resistance [38,39,40]. This strategy not only extends the effectiveness of new antibiotics but also ensures a more sustainable supply of treatments for resistant infections. In this respect, in silico tools are incredibly valuable. Techniques like molecular docking and molecular dynamics simulations would help researchers predict how potential clovibactin analogs might interact with lipid II and other targets. Molecular docking can quickly screen large libraries of compounds to find those with the best binding affinities, while molecular dynamics simulations offer detailed insights into the stability and behavior of these interactions over time. These computational methods not only speed up the discovery process but also improve the accuracy of identifying promising candidates for further testing. Using these advanced in silico techniques can accelerate the development of new lipid II-targeting antibiotics, potentially easing the ongoing challenge of antimicrobial resistance.

This study marks a significant advancement in the fight against antibiotic resistance by not only investigating clovibactin-like compounds that specifically target lipid II but also being the first to rationalize the discovery of potential antibiotics through a detailed examination of the molecular interactions between clovibactin and lipid II—an immutable target in Gram-positive bacteria. While many studies have explored various antibacterial agents, our research uniquely emphasizes lipid II as a critical focal point for developing new antibiotics. By analyzing how these compounds bind to lipid II, we aim to uncover novel candidates that can effectively combat bacteria that have developed resistance to current treatments. This targeted strategy not only enhances our understanding of clovibactin derivatives but also holds promise for creating antibiotics that are less likely to provoke resistance in bacterial populations.

Moreover, we employed cutting-edge computational methods, including molecular docking and dynamic simulations, to delve into the specifics of how these promising antibiotics interact with lipid II. This innovative approach is particularly noteworthy, as it enables us to efficiently screen a vast array of potential compounds and identify those with the strongest likelihood of success for further laboratory testing. Our findings reveal specific compounds that form stable and effective interactions with lipid II, displaying binding affinities comparable to or even exceeding those of clovibactin. By thoroughly exploring the stability and effectiveness of these interactions, our research not only provides critical insights into the development of targeted antibiotics but also underscores the significance of understanding the mechanisms behind their efficacy. Ultimately, this study represents an essential step in addressing the urgent challenge of antibiotic-resistant bacteria and paves the way for the development of more effective therapeutic options.

Our results identified five compounds that formed stable complexes with lipid II, exhibiting binding energies comparable to or greater than that of clovibactin. To gain deeper insights, molecular interaction diagrams were utilized. These findings suggest that these compounds can be tested in vitro against various bacterial strains to evaluate their potential as new antibiotics. To ensure selectivity for lipid II, we also studied the interactions of these compounds with lipid I, lipid II, lipid III, and C55PP. Leveraging these advanced in silico techniques could accelerate the development of new lipid II-targeting antibiotics, potentially easing the ongoing challenge of antimicrobial resistance.

## 2. Results and Discussion

### 2.1. Data Collection

Our primary objective was to identify new antibiotics that selectively target lipid II. To achieve this, we utilized PubChem’s similarity search protocols to identify compounds similar to clovibactin, which is already known to target lipid II. By applying the similarity criterion from PubChem, 2622 compounds were identified. After Lipinski’s rules and the ADME-TOX protocol were applied, only 25 compounds passed further analysis and are shown in Table 1 jointly with clovibactin and teixobactin. As can be seen, most of the compounds failed in levels III–IV of toxic probability. According to the globally harmonized system of labeling of chemicals (GHS). Level III substances have moderate toxicity and can cause significant adverse effects if ingested, inhaled, or in contact with the skin. The LD50 for oral exposure is 50–300 mg/kg and 200–1000 mg/kg for dermal exposure. Inhalation limits are 0.5–2.0 mg/L for dusts/mists and 2.0–10.0 mg/L for gases/vapors. They are labeled “Danger” with statements like “Toxic if swallowed”, “Toxic in contact with skin”, and “Toxic if inhaled”. Level 5 substances have low toxicity but still require caution. Their LD50 for oral and dermal exposure is 2000–5000 mg/kg, and inhalation limits are 10.0–20.0 mg/L for dusts/mists and 20.0–50.0 mg/L for gases/vapors. They are labeled “Warning” with statements like “May be harmful if swallowed”, “May be harmful in contact with skin”, and “May be harmful if inhaled. In this sense, the compounds reported here could be used as drugs at small doses with no apparent risk. Therefore, these 25 compounds were docked to the selected target [41].

### 2.2. Molecular Docking

The 25 molecules with favorable drug-likeness and ADME-TOX properties were docked against multiple targets. Initially, lipid II was considered the primary target due to clovibactin’s selective mechanism of action. However, two factors prompted the inclusion of additional targets: (1) the selectivity of clovibactin must be validated to align with experimental reports and (2) the preservation of the pyrophosphate moiety in lipid III and lipid II precursors, coupled with minimal variability in amino acid residues. Consequently, lipid I, C55P, and C55PP were selected as targets. In Gram-positive bacteria, lipid I, lipid III, undecaprenyl phosphate (C55P), and undecaprenyl pyrophosphate (C55PP) are vital for peptidoglycan biosynthesis. Lipid I forms when C55P reacts with UDP-MurNAc-pentapeptide, while lipid III helps transfer sugar units during polysaccharide assembly. C55P acts as a carrier, moving peptidoglycan precursors across the cell membrane and cycling between the phosphorylated and pyrophosphorylated states [42,43]. C55PP is then converted back to C55P, ensuring a steady supply of materials for cell wall construction [42]. These lipids are essential for the structural integrity and growth of Gram-positive bacteria, making them key targets for antibiotics. Therefore, any perturbation or interaction with these precursors disrupts bacterial cell wall synthesis, leading to bacterial death [42].

The docking results for clovibactin, teixobactin, and clovibactin-like compounds are summarized in Table 2. Clovibactin demonstrated strong binding affinities to lipid I (−9.91), lipid II (−7.61), and lipid III (−7.07), indicating high interaction with these targets. In comparison, teixobactin exhibited lower binding affinities, with the highest affinity for lipid I (−7.17), followed by lipid II (−4.25), and lipid III (−4.95). This suggests that clovibactin may have a broader and stronger interaction profile with these essential bacterial cell wall synthesis components. None of the compounds examined in this work showed affinity for C55P and C55PP, confirming their higher affinity for at least one lipid target.

Among the clovibactin-like compounds, several showed promising binding affinities. Compound 24 exhibited the strongest binding to lipid I (−7.84) and moderate binding to lipid II (−5.02), making it a potential candidate for further development. Compound 16 also showed good binding to lipid I (−7.14) and moderate binding to lipid II (−5.68), while Compound 25 demonstrated strong binding to lipid I (−7.06) and moderate binding to lipid II (−5.75). Notably, Compound 22 displayed a high binding affinity to lipid II (−7.41), surpassing that of clovibactin, which highlights its potential as a selective antibiotic.

Based on the docking results, the most promising compounds as potential antibiotics are compounds 7, 21, 22, 24, and 25. These compounds exhibit binding affinities similar to or better than those of clovibactin and teixobactin, indicating their potential as effective antibiotics targeting bacterial cell wall synthesis. Compound 24 stands out due to its strong binding to lipid I and moderate binding to lipid II, suggesting a high level of selectivity and efficacy.

Interestingly, while experimental studies have primarily suggested that clovibactin targets lipid II, our results indicate that clovibactin also has a strong binding affinity for lipid I. This dual targeting could be due to the structural similarities between lipid I and lipid II, both of which are crucial intermediates in the bacterial cell wall synthesis pathway. Clovibactin’s ability to bind to multiple lipid precursors may enhance its antibacterial efficacy by disrupting different stages of cell wall synthesis. This hypothesis is supported by studies showing that clovibactin binds to the pyrophosphate moiety of various lipid precursors, including lipid I and lipid II [44,45,46,47]. However, further experimental validation is needed to confirm these findings and to fully understand the implications of clovibactin’s binding properties.

**Figure 1:**
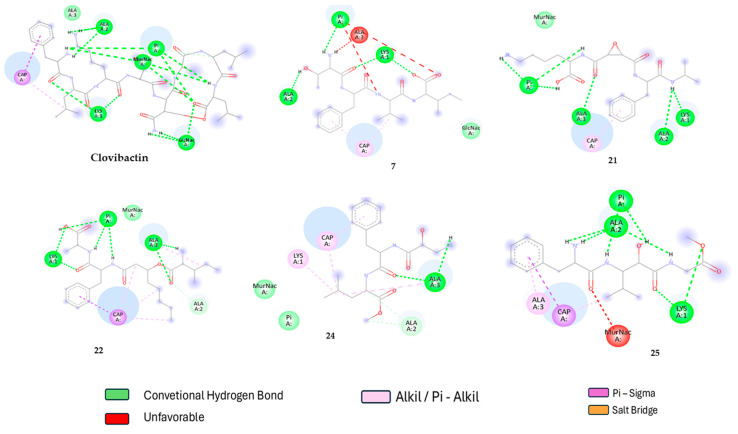
Comparative Interactions of clovibactin and compounds with lipid II: focus on hydrogen bonds involving the pyrophosphate group.

To gain a deeper understanding of the interactions that drive the formation of complexes formation with lipid II, a 2D interaction diagrams were generated by the mean of discovery studio visualizer 4.5 software (https://discover.3ds.com/discovery-studio-visualizer-download accessed on 21 December 2021). Therefore, the lipid II-ligand complexes for the best compounds were compared with clovibactin one and are shown in Figure 1.

According to Figure 1, clovibactin primarily interacts with lipid II through several strong hydrogen bonds and other Van der Waals interactions with the pyrophosphate (Pi) group; these interactions are crucial for stabilizing the binding of clovibactin to lipid II, which is essential for its antibacterial activity. In the other compounds (7, 22, 24, and 25), the dominant interactions also involve hydrogen bonding and Van der Waals interactions with the pyrophosphate group of lipid II. Additionally, the latter compounds interact with N-acetylmuramic acid (MurNac), a compound involved in peptidoglycan biosynthesis, further contributing to their antibacterial properties [42]. The strength and pattern of these interactions vary among the compounds, influencing their binding affinity and stability.

When comparing the molecular interactions of these compounds with clovibactin, compounds 21 and 22 stand out due to their strong hydrogen bonding and electrostatic interactions with the pyrophosphate group, like clovibactin. Compound 22 forms multiple hydrogen bonds with the oxygen atoms of the pyrophosphate group, which are particularly strong, enhancing its binding stability. This similarity in interaction patterns suggests that compounds 21 and 22 may have a mechanism of action comparable to that of clovibactin, potentially leading to similar antibacterial efficacy; therefore, while all the compounds exhibit significant interactions with the pyrophosphate group of lipid II, compounds 21 and 22 show the most similarity to clovibactin in terms of both the number and strength of hydrogen bonds.

### 2.3. Molecular Dynamic

We simulated the structures of all the complexes in a water environment to determine how the ligands affect their stability. We used the root mean square deviation (RMSD), a common method to measure how much a protein’s backbone shifts from its starting shape to its final position. We can understand the stability of the molecule over time by observing these shifts during the simulation. If the protein does not move much, it means that the structure is more stable and less likely to become unstable.

After the docking protocols, the assessment of the complexes’ stability over time is necessary. Therefore, the molecular dynamics for complexes between clovibactin and compounds 7, 21, 22, 24, and 25 were carried out over 100 ns.

**Figure 2:**
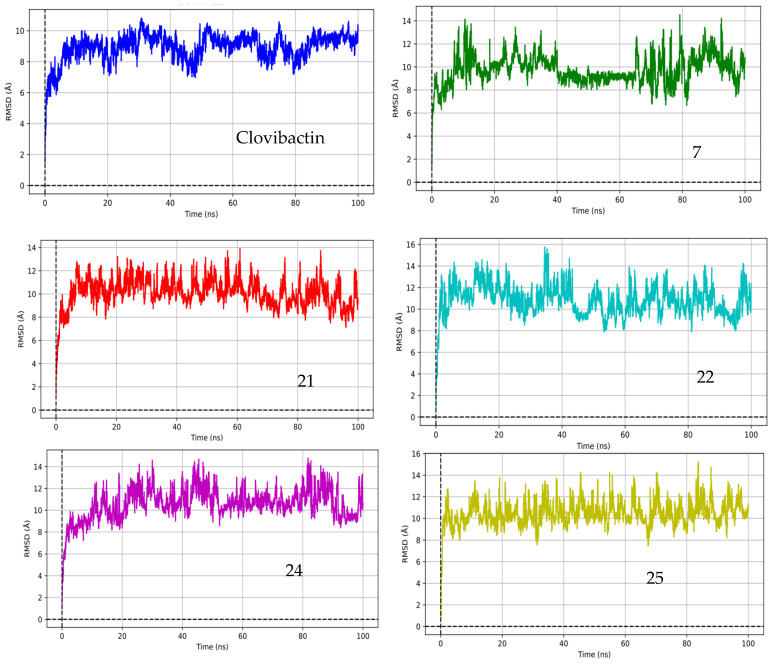
Structural stability assessment: RMSD analysis of clovibactin and its analogs in complex with lipid II.

Figure 2 illustrates the root mean square deviation (RMSD) for the lipid II-compound complexes studied in this work. The RMSD serves as an indicator of both the stability and flexibility of the complexes over the simulation period. As shown in Figure 2, clovibactin and the rest of the compounds studied in this work reach equilibrium at approximately 5 ns. Prior to this equilibration, the RMSD is 6 Å, suggesting that the system undergoes a reorganization of its molecular structure and conformation [47,48,49,50]. Given that these complexes are not conventional protein-ligand systems and considering the numerous dihedral angles in clovibactin, an RMSD higher than 4 Å is deemed acceptable [51,52].

Based on the RMSD values depicted in the figure, clovibactin demonstrates a relatively stable interaction with lipid II, with RMSD values stabilizing at around 2 Å after approximately 20 ns, indicating equilibration. Compounds 7, 21, 22, 24, and 25 exhibit varying degrees of stability. Compounds 21, 22, 24, and 25 show a stabilization pattern similar to that of clovibactin, suggesting comparable binding stability. Compound 7 exhibits higher RMSD values, indicating a few less stable interactions. Compound 24 stabilizes around 3 Å, while Compound 25 shows significant fluctuations; however, between 40 and 60 ns, it presented the smallest RMSD.

Considering that lipid II is not a protein and has several dihedral angles, and that compounds 7, 21, 22, 24, and 25 are peptides with multiple dihedral angles, these fluctuations could be attributed to these molecules’ inherent flexibility and conformational changes [51,52]. Between 40 and 60 ns, the RMSD values for most compounds exhibit minor fluctuations, potentially indicating conformational adjustments or minor binding-site rearrangements. This period likely represents a phase of dynamic equilibrium in which the compounds explore their best binding conformations.

**Figure 3:**
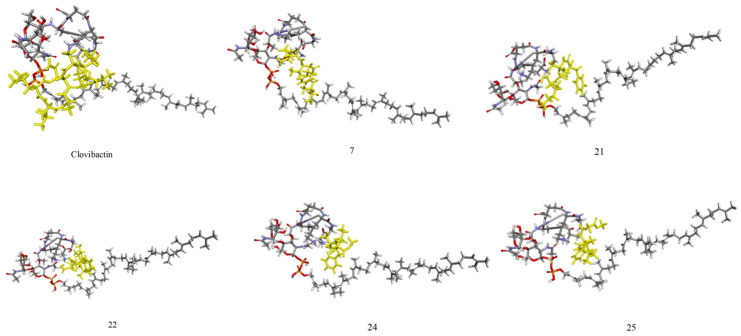
Structural insights into minimum energy complexes from 100 ns of molecular dynamics simulation.

After 60 ns, the RMSD values for clovibactin and the other compounds generally stabilize, indicating that the systems have likely reached a more stable equilibrium state. This suggests that the major conformational adjustments and binding-site explorations have been completed, and the compounds are now maintaining their interactions with lipid II with minimal fluctuations. This stabilization phase is crucial for understanding the compounds’ long-term binding stability and their potential efficacy as antibiotics. Figure 3 illustrates the lowest energy complexes between lipid II and the compounds studied herein (in yellow).

Interestingly, the fluctuations in RMSD values observed in Figure 2 correspond to variations in hydrogen bond formation between the solvent and lipid II, particularly with the pyrophosphate moiety. Under physiological conditions (pH = 7.4), pyrophosphate exists in a mixture of single and double protonation states, which favors the formation of net charges capable of acting as electron attractors or bases [53]. This establishes a competitive equilibrium between hydrogen bond formation and proton transfer among pyrophosphate, water, and ligands, specifically clovibactin and its derivatives. As ligands undergo conformational changes during simulation, this dynamic equilibrium influences their interactions and stability, highlighting the interplay between ligand conformations and the biological environment [54]. The result is that the stability and binding affinity of the ligands can be affected not only by their intrinsic properties but also by the surrounding solvent dynamics and protonation states of key molecular entities, such as pyrophosphate. Analyzing these relationships lends further insight into the conditions that dictate ligand efficacy in drug development efforts.

Notably, clovibactin forms a lattice-like structure, interacting not only with the pyrophosphate group but also with certain amino acids of lipid II. This lattice structure is akin to that observed experimentally, where it has been reported that clovibactin forms a fibril that precipitates and eradicates the bacteria [31,32]. Acccording to Figure 3, the rest of the compounds present a similar behavior with some of them interacting with the lipidic and amino acid part of the lipid II (21, 22 and 25).

**Figure 4:**
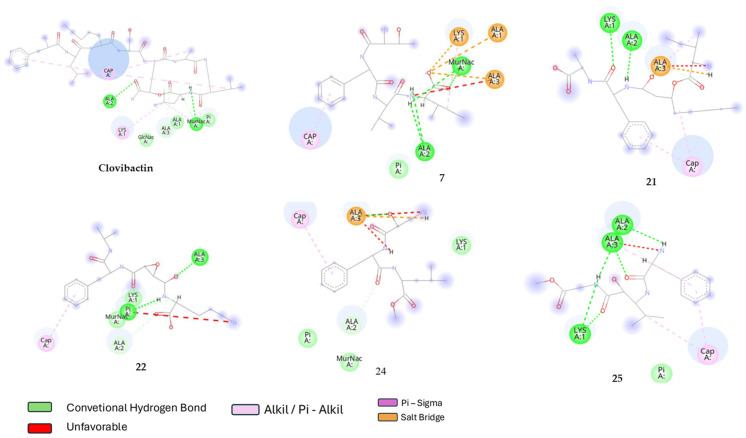
2D Interaction mapping for minimum energy complexes generated by molecular dynamics simulations.

Figure 4 presents a 2D diagram of these interactions. As previously mentioned, the primary forces driving the antibacterial activity of clovibactin are Van der Waals interactions, mainly hydrogen bonding. However, given the molecular size of clovibactin, these hydrogen bonds are not limited to the pyrophosphate group but also extend to the amino acid components. This dual interaction significantly enhances the overall binding strength. Similarly, the rest of the compounds present molecular interactions with pyrophosphate and some lipid II amino acids. Compounds 22 and 25 stand up between these compounds due to the formation of several hydrogen bonds with pyrophosphate and some other strong molecular interactions such as the saline bridge with amino acids of lipid II. Compounds 7 and 24 present several hydrogen bonds; however, no interaction with the pyrophosphate group is observed in the minimum energy pose.

The MM-PBSA approach assesses and understands the interactions and binding energies between proteins and ligands in biomolecular complexes. Ligand binding energies were estimated using molecular dynamics (MD) simulations conducted in YASARA by employing Equation 1. In this context, In YASARA structure, the macro is parameterized such that higher positive values indicate stronger binding affinity, whereas negative values do not necessarily mean that there is no binding. However, to make our results clearer and consistent with other studies, we inverted and rewrote the equation in the YASARA macro, which is now shown as Equation (1).

(1) $$Binding\;Energy=\Delta E_{Solv}+\Delta E_{pot}$$

where,

(2) $$∆E_{Solv}=Esolv_{complex}-\left(Esolv_{Recept}+Esolv_{Ligand}\right)$$

and,

(3) $$∆E_{pot}\;=\;Epot_{complex}-\left(Epot_{Recept}+Epot_{Ligand}\right)$$

The average binding energy is the sum of the change in the solvation energy (solv) and the change in the potential energy (pot) during the complexation process; both parameters must compensate for minimizing the binding energy. In this form, the results are understandable in the same line as in the specialized bibliography. Table 3 shows the parameter values used to calculate the average binding energy.

Looking at Table 3, we examined the binding energies of different compounds with lipid II, focusing on potential energy and solvation energy. Clovibactin, a newly discovered antibiotic, was used as our reference point. The binding energy, which is the sum of the potential energy and solvation energy changes, indicates the stability of the complex. A more negative binding energy indicates a more stable complex, usually indicating better biological activity.

Clovibactin had a binding energy of −16.73 kcal/mol, with a significant negative potential energy of −40.82 kcal/mol, partially balanced by a positive solvation energy of 24.09 kcal/mol. This moderately negative binding energy suggests a stable interaction with lipid II. On the other hand, Compound 7 and Compound 21 had positive binding energies of 19.77 kcal/mol and 25.45 kcal/mol, respectively, despite their strong negative potential energies. Their high positive solvation energies led to overall instability, making them less promising than clovibactin.

Compound 22 was the most promising candidate, with a binding energy of −25.50 kcal/mol. This highly negative binding energy was mainly due to an extremely negative potential energy of −244.50 kcal/mol, only partially offset by a positive solvation energy of 218.99 kcal/mol. The significant stability of this complex suggests a strong potential for biological activity, making Compound 22 a top candidate for further development as an antibiotic.

So far, while clovibactin serves as a good reference with moderate stability, Compound 22 shines due to its superior binding affinity, indicated by its highly negative binding energy. Compound 25, with a binding energy of −5.96 kcal/mol, also shows potential for biological activity. This binding energy comes from a negative potential energy of −22.38 kcal/mol and a positive solvation energy of 16.43 kcal/mol. Although not as stable as Compound 22, Compound 25’s negative binding energy suggests that it could still be a viable candidate for further development as an antibiotic. Further experimental validation is needed to confirm these findings and to assess the biological activities of both Compound 22 and Compound 25. Compounds 7, 21, and 24, with their positive binding energies, appear less stable and thus less likely to have significant biological activity.

The relationship between the potential energy (Eopt) and solvation energy (Esolv) can provide insights into the mechanism of action of compounds like clovibactin, particularly in the context of fibril formation. Clovibactin’s mechanism of action involves binding to lipid II and disrupting cell wall synthesis, which can lead to the formation of fibrils that aggregate and exert antibacterial effects.

A highly negative potential energy (Eopt) indicates strong interactions between the compound and lipid II, which are crucial for the initial binding and stability of the complex. This strong binding can facilitate the aggregation of lipid II molecules, thereby promoting fibril formation. On the other hand, the solvation energy (Esolv) reflects the compound’s interaction with the solvent, which can influence the overall stability and solubility of the complex. A positive solvation energy can partially offset the binding affinity; however, if the potential energy is sufficiently negative, the complex remains stable [55,56,57,58].

In our analysis, we observed that certain compounds exhibited elevated solvation energy compensation, which could be attributed to their structural characteristics and interactions with the solvent environment. For instance, compounds with more hydrophilic functional groups tend to have higher solvation energies due to the increased solvation shell formation around these regions [57,58]. However, this increase in solvation energy can be offset by stronger interactions at the binding site, resulting in favorable binding affinities.

According to Table 3, the binding energy in this study appears to be driven by the potential energy, as all ∆E_Solv values are positive. This indicates that compounds with more negative ∆E_pot values, rather than ∆E_Solv, will be more active. As defined by Equation (2), the solvation energy of the complex must be more negative than the sum of the individual components, meaning that the solvation capacity of the complex drives this process. Although solvation is complex, in this study, it can be rationalized in terms of the molecular volume; larger molecular volumes generally result in higher solvation energies [57,58]. Except for clovibactin, the analogs studied here are linear dipeptides, with Compound 22 being the only one featuring an alkyl moiety in its structure. This characteristic confers a higher molecular volume (due to its rotatability) and makes it more challenging to solvate.

Scientific studies have shown that the balance between binding affinity and solvation effects is critical for forming stable fibrils and for the antibacterial efficacy of compounds targeting lipid II [59,60,61]. Therefore, compounds with highly negative potential energies and manageable solvation energies, like Compound 22, are promising candidates for further investigation as potential antibiotics. However, further experimental validation is needed to confirm these findings and to assess the biological activities of both Compound 22 and Compound 25. Compounds 7, 21, and 24, with their positive binding energies, appear less stable and thus less likely to exhibit significant biological activity.

**Figure 5:**
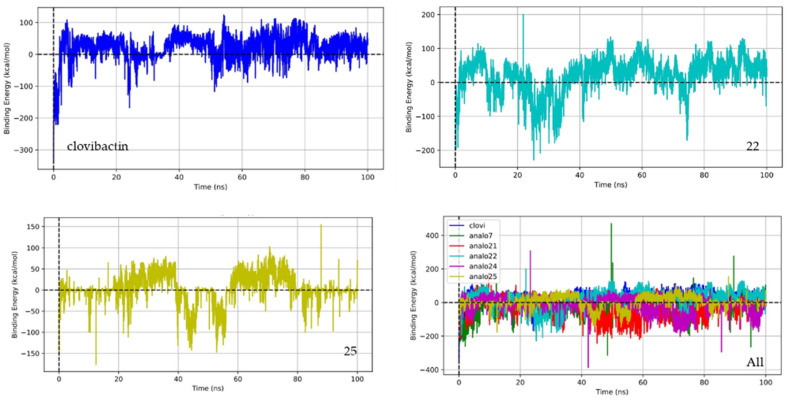
Dynamics of binding energy for clovibactin, Compound 22, and Compound 25 with lipid II throughout the simulation.

To gain more insight into the binding energy variation during the simulation, the binding energy trajectories for clovibactin, Compound 22, and Compound 25 are depicted in Figure 5. This figure provides important insights into their stability and interaction dynamics with lipid II.

Clovibactin shows a relatively stable binding energy throughout the simulation, with minor fluctuations around its average value of −16.73 kcal/mol. This result confirms consistent interactions with lipid II, suggesting that clovibactin maintains a strong and steady binding affinity. The minor fluctuations observed are typical in molecular dynamics simulations and reflect the natural dynamic behavior of the molecular complex. In contrast, Compound 22 exhibits a more negative binding energy trajectory, averaging around −25.50 kcal/mol, indicating a stronger binding affinity compared to clovibactin. The trajectory of Compound 22 shows more pronounced fluctuations, reflecting its dynamic interactions with lipid II and this compound’s conformation change along the simulation. These fluctuations could be due to the compound’s ability to form multiple stable interactions or its higher flexibility in binding modes, which can enhance its overall binding affinity. Despite these fluctuations, the overall binding energy remains highly negative, suggesting a very stable complex. Compound 25, with a binding energy trajectory averaging around −5.96 kcal/mol, shows less negative binding energy, indicating a weaker binding affinity than clovibactin and Compound 22. The trajectory of Compound 25 may exhibit larger fluctuations, indicating less stable interactions with lipid II. These fluctuations could be due to weaker or fewer binding interactions, which would render the complex less stable over time. In summary, while clovibactin maintains a stable binding profile, Compound 22 demonstrates superior binding affinity with dynamic interactions, and Compound 25 appears to be the least stable, with the weakest binding affinity among the three.

Analog 22 possesses unique binding characteristics that ensure strong interactions with lipid II, which are crucial for its effectiveness as an antibiotic. Among these features, the presence of phenyl and pentyl groups—both nonpolar—stands out, as they confer significant stability to the ligand through Pi-Sigma interactions with the undecaprenyl lipid portion of lipid II. This strong interaction is complemented by polar interactions, particularly hydrogen bonds between different amino groups and the pyrophosphate group of lipid II, an immutable target, which provides additional binding strength to the ligand-target interaction. Furthermore, these unique binding properties may play a pivotal role in mitigating the emergence of bacterial resistance. By demonstrating a high binding affinity and engaging in stable, multifaceted interactions, Compound 22 not only enhances its antimicrobial efficacy but also creates substantial barriers to resistance development. The ability to occupy critical binding sites and the potential to induce conformational changes in the target make it more challenging for bacteria to adapt, emphasizing that developing antibiotics with such characteristics would be highly beneficial for long-term therapeutic success.

These results provide strong evidence that compounds 22 and 25 are good candidates to become potential antibacterial compounds. However, in silico methodologies, while invaluable for guiding the drug discovery process, do present several constraints that must be acknowledged in the context of experimental validation. One significant limitation is the reliance on computational models, which may oversimplify biological systems, leading to discrepancies between the predicted and actual interactions. These models often depend on existing structural data, which may not encompass the full diversity of conformational states present in real biological environments. Additionally, in silico approaches cannot accurately predict the dynamic behavior of biological molecules under various physiological conditions, potentially resulting in misleading conclusions regarding efficacy and binding affinity. Consequently, while in silico studies can streamline the initial phases of drug design and identification, it is imperative to complement these findings with thorough experimental investigations. This hybrid approach ensures that the limitations of in silico predictions are adequately addressed, providing a comprehensive understanding of compound behaviors in biological systems.

## 3. Materials and Methods

### 3.1. Data Collection

First, the molecular structures of clovibactin and lantibiotics targeting lipid I, lipid II, lipid III, C55P, and C55PP for Gram-positive bacteria were retrieved from PubChem. Since the main goal of this work was to find new potential structures of clovibactin-Like, a similarity search was conducted using the 2D-similarity PubChem algorithm. PubChem’s 2-D similarity method uses a technique called atom-centered Gaussian-shape comparison, which is implemented in the Rapid Overlay of Chemical Structures (ROCS) tool. This method aligns the 2-D structures of two molecules to maximize their overlap, effectively comparing their shapes. The similarity is measured using three metrics: Shape-Tanimoto (ST) for steric shape similarity, Color-Tanimoto (CT) for the overlap of functional groups like hydrogen bond donors and acceptors, and Combo-Tanimoto (ComboT), which combines the ST and CT scores [62,63]. Consequently, a list of 2624 compounds was retrieved and saved.

### 3.2. Minimum Energy Structures

The structures of clovibactin, teixobactin, and the target molecules lipid I, lipid II, lipid III, FC55P, and FC55PP, along with the 2624 clovibactin-like compounds identified through PubChem searches, were obtained through a two-step process. First, the minimum energy conformer was determined using the Avogadro 2.1 software [64] by employing a genetic algorithm for energy minimization. The resulting conformer was saved as a .pdb file and further refined using Gaussian 09 for Linux by applying the PM6 semi-empirical method, while considering the reported stereochemistry. The final minimized structures were saved as .pdb files and were subsequently used in the docking procedure.

### 3.3. ADME-Tox Properties

ADME-Tox properties refer to the absorption, distribution, metabolism, excretion, and toxicity characteristics of a compound. These properties are crucial in drug discovery and development because they determine how a drug behaves in the body and its potential safety profile [65]. Calculating the ADME-Tox properties of peptides is essential because these properties determine how a potential therapeutic agent behaves in the body, including its safety profile. Peptides often have unique characteristics in terms of absorption, distribution, metabolism, excretion, and toxicity due to their size and structure, which can significantly impact their effectiveness and safety as drugs [66]. By evaluating these properties early, researchers can identify and optimize peptides with favorable ADMET profiles, thereby reducing the risk of late-stage failures in drug development [67,68].

Filtering datasets based on ADME-Tox properties is equally important before conducting further assessments, such as in vitro assays and molecular dynamics screening. This preliminary filtering helps to eliminate compounds with poor pharmacokinetic or toxicological profiles, allowing researchers to focus on the most promising candidates. In addition, this approach enhances the efficiency of the drug discovery process by reducing the number of compounds that need to be tested experimentally, thereby reducing both time and costs. Moreover, it increases the likelihood of identifying viable drug candidates for clinical trials [69,70,71]. In this sense, clovibactin-like structures were filtered using ProTox 3.0 [68] and compared with the ADMET properties of both clobivactin and teixobactin. In every instance, the SMILES code was entered into the ProTox, and the resulting data was downloaded as a .csv file. These data were then compared to the properties of clovibactin. ProTox 3.0 assesses a compound’s safety using several key criteria.

In our analysis of the ADME-Tox properties using ProTox 3.0 software, we established specific toxicity level cutoffs based on the guidelines provided by the globally harmonized system for classification and labeling of chemicals (GHS), as well as pertinent literature in the field of drug discovery. Compounds with an LD50 value exceeding 5000 mg/kg were designated as non-toxic, while values between 2000 and 5000 mg/kg indicated a need for caution. These thresholds were selected to ensure that the safety profiles of potential therapeutic candidates align with established standards in drug development [71,72]. Additionally, we compared our findings with clinical thresholds established in pharmaceutical research to validate the appropriateness of our cutoff criteria [71]. This comparison allows us to ensure that the selected compounds not only exhibit favorable safety characteristics but also meet the rigorous expectations typically required in clinical settings [71,72]. By utilizing these criteria, we aim to enhance the reliability of our ADME-Tox profiling, significantly reducing the risk of pursuing compounds that may fail due to safety concerns during subsequent stages of development.

### 3.4. Molecular Docking

Molecular docking is a computational technique used to predict interactions between two molecules, typically a small molecule (ligand) and a protein (receptor). This method is extensively utilized in drug discovery and development to identify potential drug candidates by simulating and analyzing the molecular fit and binding affinity. Furthermore, molecular docking is a versatile and powerful tool that significantly enhances drug discovery and repurposing efficiency and effectiveness; by accurately predicting the interactions between drugs and their targets, it aids in identifying promising candidates, elucidating their mechanisms of action, and discovering new therapeutic applications for existing drugs [73,74]. In this work, the molecular interactions between clovibactin and teixobactin, as well as the filtered clovibactin-like compounds, were studied employing molecular docking using both autodock-vina 2.0 and AutoDockGPU 1.0 software [75]. It is important to point out that autodock-vina has been used before in the study of peptide-ligand and protein interactions in some works [76,77,78]. Nevertheless, AutoDockGPU jointly with ADADELTA showed better performance in this type of system; therefore, all of the results reported herein have been carried out using auto dock [79].

To enhance our docking studies and gain a deeper understanding of antibiotic interactions, we included additional lipid targets—lipid I, lipid III, C55P, and C55PP—alongside lipid II. Each of these lipids is crucial for the biosynthesis of the bacterial cell wall, making them relevant targets for effective antibiotics [78]. Lipid I acts as a precursor in the peptidoglycan synthesis pathway, while lipid III is key in transferring sugar units during polysaccharide assembly. Additionally, C55P and C55PP are essential for transporting peptidoglycan precursors across the bacterial membrane [78]. By evaluating binding affinities across these multiple targets, we aim to identify compounds with potential multi-target activity, significantly enhancing their therapeutic efficacy. This multi-target approach is particularly beneficial for combating antibiotic resistance, as it allows for the development of compounds that can disrupt several critical pathways, thereby increasing the likelihood of effective bacterial inhibition. The affinity of the antibiotics was assessed by comparing the mean docking scores obtained for each target.

#### 3.4.1. The Preparation of Ligands and Targets

The ligands were first prepared by cleaning to remove unwanted molecules or ions. Subsequently, Gasteiger charges were calculated and assigned to the ligands to ensure accurate electrostatic interactions during docking. The prepared ligands were then stored in the. pdbqt format, which is compatible with autodock vina´s softwares.

A similar preparation procedure was applied to the target molecules, including lipid I, lipid II, lipid III, C55P, and C55PP. Each target was cleaned to remove any extraneous molecules, and Gasteiger charges were added to ensure proper interaction modeling. These targets were also saved in the .pdbqt format for consistency and compatibility with the docking studies.

#### 3.4.2. Docking Protocol

For molecular docking studies, both ligands (antibiotics and clovibactin-like compounds) and targets (Lipid I, lipid II, lipid III, C55P, and C55PP) were prepared in the PDBQT format. Even with the inherent flexibility of lipids, we opted to treat lipids as a rigid entity during the simulations. This decision was based on the structural context of lipid II, which is firmly anchored within the bacterial lipid bilayer, thereby restricting its mobility. Additionally, given that both clovibactin and its analogs primarily interact with the pyrophosphate group and the pentapeptide of lipid II, the impact of flexibility is largely confined to the movement of the peptide bonds. This approach is supported by previous studies [76,77,78], which discuss similar methodologies in the context of lipid interactions. We consider this simplification appropriate for the initial stages of our study, focusing on binding affinities.

The box size and box-center coordinates for each target are detailed in Table 4. The iterative docking process was then initiated using a custom script with AutoDockGPU [75]. The ADADELTA search method, a first-order gradient-based local optimization algorithm, was employed [79]. For each docking run, 100 distinct conformations were generated, with a maximum of 42,000 generations and 2,500,000 evaluations per Local Genetic Algorithm (LGA) calculation.

### 3.5. Molecular Dynamics

The stability of the ligand-receptor complex is essential for predicting how well potential drug candidates will bind to their targets. Molecular dynamics (MD) simulations enable researchers to observe the behavior of these complexes under physiological conditions, offering valuable insights into the strength and duration of their interactions. This information is crucial for optimizing drug design, as it helps in selecting compounds with the most favorable binding properties. Additionally, by simulating the dynamic environment of the target, MD simulations can identify potential issues related to drug resistance, such as mutations in the target protein, which may affect the stability and binding of the drug. Overall, the ability to study the stability of ligand-receptor complexes over time makes MD simulations an invaluable tool for the rational design and development of new therapeutics [80].

After filtering the results by ADME-TOX calculation along with the scoring values on the molecular docking, the stability of the best scoring complexes was assessed by employing molecular dynamics calculation. To achieve this goal, the software YASARA Dynamics 32.12.24 and the AMBER14 force field were used [81].

For the molecular dynamics simulations, the complexes of the selected ligands with their respective targets were loaded and placed in a cubic box with an extension of 20 Å around the molecule using periodic boundary conditions to enhance the accuracy of the simulations. The simulation conditions were set to reflect physiological parameters: pH of 7.4, sodium chloride concentration of 0.9% to neutralize the system, temperature of 298 K, and water density of 0.997 g/mL. The pressure was regulated by adjusting the volume of the simulation box according to the water density, thereby simulating the pressure conditions that the bacterial structure would experience in a human physiological environment. All simulations were conducted with a limit of 100 ns for each analysis. After the simulations were conducted, they were analyzed using a YASARA macro (md_analyze_dynamics.mcr). This macro allows the output of several pieces of information, such as root mean square deviation (RMSD). Finally, using the macro (md_analyzebindenergy.mcr), the binding energies were computed using the adaptive Poisson-Boltzmann Solver, implemented already in the macro (Equation (1)) with a little modification such as converge with the universal meaning of binding energy (Equation (1)): A more negative binding energy indicates a more stable complex, which correlates with greater potential efficacy of the drug. This measurement allows us to compare the likely effectiveness of different drug candidates, including their relative performance against established compounds like clovibactin and teixobactin.

## 4. Conclusions

In this study, we searched for clovibactin-like compounds using specific search criteria established in the PubChem database. We filtered similar compounds using ProTox 3.0 software to assess their ADME-Tox properties. This initial step was crucial, as it allowed us to eliminate compounds with poor pharmacokinetic profiles and potential toxicity, ensuring that we focused on the most promising candidates for further investigation. We then employed molecular docking protocols to explore how these selected compounds interacted with lipid II, a vital target in bacterial cell wall synthesis. Our docking results highlighted Compound 22 as a standout candidate, showcasing a remarkably strong binding affinity. The principal molecular interactions observed suggest that this compound forms stable complexes with lipid II, which is essential for its potential effectiveness as an antibiotic. Furthermore, we examined the binding energies of these compounds, providing valuable insights into their interactions. Compound 22 exhibited a highly negative binding energy of −25.50 kcal/mol, indicating a strong affinity for lipid II. We also considered the influence of solvation energy and potential energy on these interactions. A balance between these energies is critical; while a highly negative potential energy suggests strong binding, the solvation energy can impact the overall stability of the complex. Understanding this balance is key to predicting how well these compounds might perform in a biological setting. While our findings are promising, it is important to emphasize that experimental validation is necessary to confirm the efficacy and safety of these compounds as potential antibiotics.

## Figures and Tables

**Table 1:** The 25 compounds passed the drug-likeness and ADME-TOX test.

Compound	Iso-SMILES	Tox-Level	CID Pubchem
Clovibactin	C[C@H]1C(=O)N[C@H](C(=O)N[C@H](C(=O)O[C@H]([C@H](C(=O)N1)NC(=O)[C@H](CO)NC(=O)[C@@H](CCCCN)NC(=O)[C@@H](CC(C)C)NC(=O)[C@H](CC2=CC=CC=C2)N)C(=O)N)CC(C)C)CC(C)C	4	146342518
Teixobactin	CC[C@H](C)[C@H]1C(=O)O[C@H]([C@H](C(=O)N[C@H](C(=O)N[C@H](C(=O)N1)C[C@H]2CN=C(N2)N)C)NC(=O)[C@H](CO)NC(=O)[C@H]([C@@H](C)CC)NC(=O)[C@@H]([C@@H](C)CC)NC(=O)[C@@H](CCC(=O)N)NC(=O)[C@H](CO)NC(=O)[C@H]([C@@H](C)CC)NC(=O)[C@@H](CC3=CC=CC=C3)NC)C	4	86341926
1	CC(C(C(=O)NC(CC1=CC=CC=C1)C(=O)O)NC(=O)C(CC(=O)N)NC(=O)CN)O	4	18485869
2	CCCC[C@H](C)[C@@H]1CC(=O)N[C@H](C(=O)N[C@H](C(=O)N[C@@H](C(=O)O1)CC(=O)O)C)CC2=CC=CC=C2	3	70881873
3	C1CCC(CC1)NC(=O)[C@H](CC2=CC=CC=C2)NC(=O)C3C(O3)C(=O)N[C@@H](CCCCN)C(=O)O	3	88197590
4	CC(C(C(=O)NC(CC(=O)N)C(=O)NC(CC1=CC=CC=C1)C(=O)NC(C(C)O)C(=O)O)N)O	4	18746171
5	CC[C@H](C)[C@@H](C(=O)O)NC(=O)[C@H](CO)NC(=O)[C@H](CC1=CC=CC=C1)NC(=O)[C@H](CC(=O)O)N	4	16122630
6	CC(C)C[C@@H](C(=O)N[C@@H](CC(=O)N)C(=O)N[C@@H](CC1=CC=CC=C1)C(=O)O)NC(=O)[C@H](CO)N	4	16741160
7	CCC(C)C(C(=O)O)NC(=O)C(C(C)C)NC(=O)C(CC1=CC=CC=C1)NC(=O)C(C(C)O)N	4	18750676
8	CCCC[C@H](C)[C@@H]1CC(=O)N[C@H](C(=O)N[C@H](C(=O)N[C@@H](C(=O)O1)[C@H](C)O)C)CC2=CC=CC=C2	3	101371349
9	CC(C(C(=O)O)NC(=O)C(CC(=O)N)NC(=O)C(CC1=CC=CC=C1)NC(=O)C(CO)N)O	4	18742358
10	CCC(C)C(C(=O)O)NC(=O)C(C(C)O)NC(=O)C(CC1=CC=CC=C1)NC(=O)C(CO)N	4	18742630
11	C1=CC=C(C=C1)C[C@@H](C(=O)NCC(=O)OCC=O)NC(=O)[C@H](CO)NC(=O)[C@H](CC(=O)O)N	3	11081110
12	CC(C)C(C(=O)O)NC(=O)C(CC(=O)N)NC(=O)C(CC1=CC=CC=C1)NC(=O)C(CO)N	4	18742361
13	C[C@H]([C@@H](C(=O)N[C@@H](CC1=CC=CC=C1)C(=O)OC)NC(=O)CNC(=O)[C@H](CC(=O)O)N)O	5	91975530
14	CCNC(=O)[C@H](CC1=CC=CC=C1)NC(=O)C2C(O2)C(=O)N[C@@H](CCCCN)C(=O)O	3	88197746
15	CC(C(C(=O)NC(CC1=CC=CC=C1)C(=O)O)NC(=O)C(CC(=O)N)NC(=O)C(C)N)O	4	18233707
16	CC(C)C[C@@H]([C@@H](C(=O)N[C@@H](C(C)C)C(=O)N[C@@H](CC1=CC=CC=C1)C(=O)OC)O)N	5	44370882
17	CNC(=O)[C@H](CC1=CC=CC=C1)NC(=O)C2C(O2)C(=O)N[C@@H](CCCCN)C(=O)O	3	88197044
18	CC(C)CC(C(=O)N[C@@H](CC1=CC=CC=C1)C(=O)OC(C)C)[C@](C(=O)N)(O)OC	5	69037115
19	C1=CC=C(C=C1)C[C@@H](C(=O)N)NC(=O)C2C(O2)C(=O)N[C@@H](CCCCN)C(=O)O	3	88197052
20	C[C@H]([C@@H](C(=O)N[C@@H](CC1=CC=CC=C1)C(=O)O)NC(=O)[C@H](CC(C)C)NC(=O)CCN)O	4	10225861
21	CC(C)NC(=O)[C@H](CC1=CC=CC=C1)NC(=O)C2C(O2)C(=O)N[C@@H](CCCCN)C(=O)O	4	88197600
22	CCCCC[C@H](CC(=O)N[C@@H](CC1=CC=CC=C1)C(=O)N[C@@H](C)C(=O)O)OC(=O)[C@@H]([C@@H](C)CC)N	3	11663205
23	CC(C(C(=O)NC(CC1=CC=CC=C1)C(=O)O)NC(=O)C(CC(=O)N)N)O	4	18219295
24	CC(C)C[C@@H](C(=O)OC)NC(=O)[C@H](CC1=CC=CC=C1)NC(=O)[C@H](CN)O	5	129012247
25	CC(C)[C@@H](C(C(=O)NCC(=O)OC)O)NC(=O)[C@H](CC1=CC=CC=C1)N	5	101177680

**Table 2:** Docking Scores of clovibactin, teixobactin, and clovibactin-like compounds against various lipid targets.

Compound	C55P	C55PP	Lipid I	Lipid II	Lipid III
Clovibactin	−1.89	−2.57	−9.91	−7.61	−7.07
Teixobactin	0.94	0.31	−7.17	−4.25	−4.95
1	−2.29	−0.82	−5.39	−5.76	−3.99
2	−2.58	−1.66	−4.99	−5.69	−3.18
3	−2.79	−2.2	−6.44	−5.83	−5.30
4	−1.54	−1.14	−5.14	−4.58	−4.12
5	−0.18	0.11	−2.65	−4.79	−3.20
6	−1.29	−1.11	−5.91	−5.13	−5.04
7	−1.4	−0.93	−4.99	−6.10	−4.73
8	−2.95	−2.43	−6.74	−5.20	−3.88
9	−1.04	−1.22	−5.38	−4.09	−4.66
10	−1.15	−0.93	−3.26	−5.71	−4.05
11	−1.01	−0.18	−4.19	−4.41	−2.97
12	−1.21	−0.59	−4.82	−4.24	−4.90
13	−0.95	0.35	−4.06	−3.90	−4.63
14	−2.02	−2.49	−4.81	−4.87	−3.94
15	−1.71	−2.44	−5.11	−5.48	−4.4
16	−2.41	−3.11	−7.14	−5.68	−5.66
17	−2.65	−2.36	−5.00	−4.99	−4.98
18	−2.34	−2.17	−5.59	−5.36	−3.25
19	−2.40	−2.87	−5.40	−5.40	−4.36
20	−1.55	−0.70	−4.88	−4.82	−5.45
21	−2.61	−2.61	−5.28	−6.08	−4.6
22	−1.37	−0.99	−4.78	−7.41	−4.91
23	−1.76	−1.74	−5.58	−4.82	−4.61
24	−3.55	−3.28	−7.84	−5.02	−6.45
25	−2.58	−2.63	−7.06	−5.75	−6.06

**Table 3:** Average ligand binding energy, change in potential, and solvation energy for complexes between compounds and lipid II.

Complex Compound-Lipid II	Binding Energy (kcal/mol)	ΔE Potential (kcal/mol)	ΔE Solvation (kcal/mol)	Molecular Volume (Bohr^3^/mol)
Clovibactin	−16.73	−40.82	24.09	4000.23
7	19.77	−46.29	66.06	3752.27
21	25.45	−100.15	125.61	4259.55
22	−25.50	−244.50	218.99	4392.21
24	4.85	−41.84	46.69	3466.97
25	−5.96	−22.38	16.43	3088.73

**Table 4:** Box size and box Cartesian coordinates for each target study in this work.

Lipid	X Center	X Size	Y Center	Y Size	Z Center	Z Size
C55PP	−11.761	50	1.152	50	1.231	48
Lipid I	−5.019	50	1.152	66	9.083	78
Lipid II	−11.577	60	−8.455	68	−4.373	62
Lipid III	−14.81	50	−13.122	66	−7.562	40

## Data Availability

Data are contained within the article.
